# Host and Cropping System Shape the *Fusarium* Population: 3ADON-Producers Are Ubiquitous in Wheat Whereas NIV-Producers Are More Prevalent in Rice

**DOI:** 10.3390/toxins10030115

**Published:** 2018-03-08

**Authors:** Meixin Yang, Hao Zhang, Xiangjiu Kong, Theo van der Lee, Cees Waalwijk, Anne van Diepeningen, Jin Xu, Jingsheng Xu, Wanquan Chen, Jie Feng

**Affiliations:** 1 State Key Laboratory for Biology of Plant Diseases and Insect Pests, Institute of Plant Protection, Chinese Academy of Agriculture Sciences, Beijing 100193 China; yyangmeixin@126.com (M.Y.); zhanghao@caas.cn (H.Z.); xjkong0528@126.com (X.K.); jinxu@ippcaas.cn (J.X.); jsxu@ippcaas.cn (J.X.); 2 Wageningen Plant Research, P.O. Box 16, 6700 AA Wageningen, The Netherlands; theo.vanderlee@wur.nl (T.v.d.L.); cees.waalwijk@wur.nl (C.W.); anne.vandiepeningen@wur.nl (A.v.D.)

**Keywords:** cropping system, Fusarium head blight, chemotype, rice stubble

## Abstract

In recent years, Fusarium head blight (FHB) outbreaks have occurred much more frequently in China. The reduction of burning of the preceding crop residues is suggested to contribute to more severe epidemics as it may increase the initial inoculum. In this study, a large number of *Fusarium* isolates was collected from blighted wheat spikes as well as from rice stubble with perithecia originating from nine sampling sites in five provinces in Southern China. *Fusarium asiaticum* dominated both wheat and rice populations, although rice populations showed a higher species diversity. Chemotype analysis showed that rice is the preferred niche for NIV mycotoxin producers that were shown to be less virulent on wheat. In contrast, 3ADON producers are more prevalent on wheat and in wheat producing areas. The 3ADON producers were shown to be more virulent on wheat, revealing the selection pressure of wheat on 3ADON producers. For the first time, members of the *Incarnatum*-clade of *Fusarium* *Incarnatum*-*Equiseti* Species Complex (FIESC) were found to reproduce sexually on rice stubble. The pathogenicity of FIESC isolates on wheat proved very low and this may cause the apparent absence of this species in the main wheat producing provinces. This is the first report of the *Fusarium* population structure including rice stubble as well as a direct comparison with the population on wheat heads in the same fields. Our results confirm that the perithecia on rice stubble are the primary inoculum of FHB on wheat and that cropping systems affect the local *Fusarium* population.

## 1. Introduction

Fusarium head blight (FHB) is a devastating disease of wheat (*Triticum aestivum* L.), barley (*Hordeum vulgare* L.) and other small grains worldwide [1,2]. In these crops FHB causes reductions in yield and grain quality, and the harvested grains are often contaminated with mycotoxins, such as trichothecenes. Consumption of these grains and products might have pernicious effects on human and animal health [3].

*Fusarium graminearum* species complex (FGSC) consists of at least 16 phylogenetically distinct species [4]: *F. graminearum* is the most widely distributed species and occurs in most FHB areas around the world [5], while *F. asiaticum* is the main FHB pathogen present in Asia [2,6,7]. Members of FGSC produce different trichothecenes [8]: NIV and acetylated derivatives (NIV chemotype), DON and primarily 3-acetyldeoxynivalenol (3ADON chemotype), or DON and primarily 15-acetyldeoxynivalenol (15ADON chemotype). These chemotypes may affect species or population ecology because the corresponding mycotoxins differ in toxicity and bioactivity [9]. A number of chemotype shifts were observed in different continents in recent years [2,10,11]. Moreover, in China, 3ADON producers were shown to have higher fitness than NIV producers as pathogens of FHB on wheat [2].

FHB pathogens usually overwinter on infested crop residues (corn stalks, rice and wheat straw, and other host plants). In spring, perithecia form on the surface of these residues, and ascospores released from perithecia on this crop debris serve as the primary inoculum of FHB. Hence, the presence of crop debris is essential for the overwintering of *Fusarium*. In agreement with this hypothesis, several studies have found a strong association between the distribution of FGSC species and cropping systems (host preference). *F. asiaticum* dominated in regions where rice is grown in rotation with wheat, as can be found in China and Korea, whereas *F. graminearum* was more common in wheat-maize rotation systems [2,12]. A similar situation was observed in the USA [13], while in Brazil, *F. graminearum* was found to be dominant in wheat [14], *F. asiaticum* in rice [15], and *F. meridionale* was determined as the predominant FGSC species on maize ears and stalks [16]. In South Africa, *F. boothii* was shown to be the best colonizer and mycotoxin producer on maize whereas *F. graminearum* was predominant on wheat [17,18]. Similar results were found in China, where *F. boothii* was prominent when maize is cultivated without rotation with wheat or rice [19]. However, nearly all studies in different agroecosystems focus on the pathogenic stage of *Fusarium* and there are few reports on the saprophytic stage on crops residues, which is crucial to the lifecycle and epidemiology of the pathogen. So far, there is no direct evidence for the overlap between *Fusarium* populations from wheat heads and the previous crop debris, which is important to understand the epidemiology of this pathogen.

In China, wheat-rice rotation within one year is the predominant cropping system in the regions along the Yangtze River, where FHB outbreaks are very frequent [2,20]. Abundant perithecia can be found on rice stubble in wheat fields in early April. Although they are usually thought to be the inoculum of FHB, the species and chemotype composition of this population are largely unknown. To study the role of the saprophytic phase of the lifecycle as a selective bottleneck, we collected a large number of *Fusarium* isolates from both wheat heads and rice stubble that were sampled from the same fields in five provinces along the Yangtze River. These populations were characterized at the species and chemotype level and their composition was compared. Subsequently, the pathogenicity of *Fusarium* species from wheat and from rice towards wheat was determined to identify possible differences in this host. Finally, these findings are discussed in the context of rice populations as the main primary inocula of FHB and the selection of wheat vs. rice on the different chemotypes of FGSC with respect to the different cropping systems.

## 2. Results

### 2.1. Fusarium Species Determination

**Figure 1:**
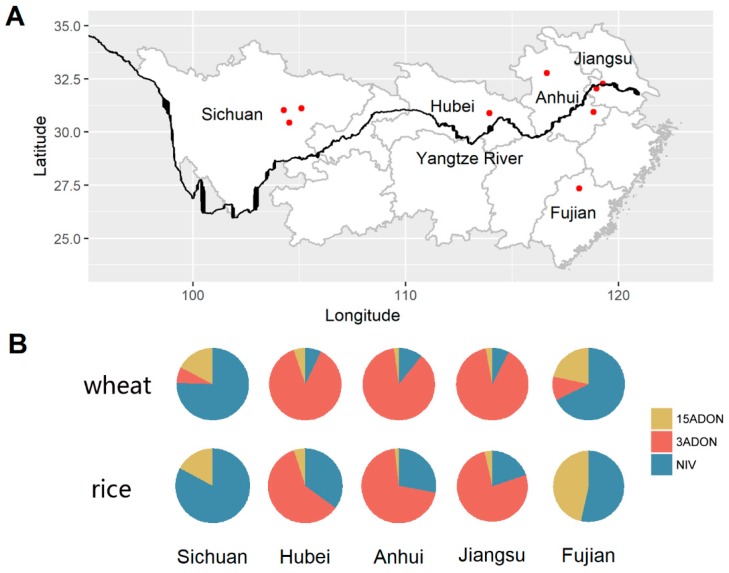
(**A**) Map of Southern China indicating the nine sampling sites in five provinces. (**B**) Trichothecenes chemotypes composition of wheat and rice populations in each province.

A total of 1246 single spore strains were isolated from nine sampling sites in five provinces (Figure 1A), 702 from rice stubble and 544 from wheat kernels. Among them, 1186 isolates could be analyzed by the MLGT assay and they belonged to three species within FGSC: *F. asiaticum* (*n* = 1110), *F. graminearum* (*n* = 67), and *F. meridionale* (*n* = 9). Based on partial *TEF-1α* gene sequences, the remaining 60 strains were identified as 10 species including *F. incarnatum*-*F. equiseti* Species Complexes (FIESC) (*n* = 30), *F. acuminatum* (*n* = 1), *F. tricinctum* (*n* = 1), *F. armeniacum* (*n* = 6), *F. kyushuense* (*n* = 2), *F. fujikuroi* (*n* = 7), *F. proliferatum* (*n* = 6), *F. concentricum* (*n* = 3), *F. nygamai* (*n* = 3), and *F. verticillioides* (*n* = 1). The distribution of these isolates is summarized in Table 1. The species composition on wheat is simple, although eight species were identified, as 93.6% of the isolates belong to *F. asiaticum*. In the rice population, we found more species (12 species) than on wheat, but again *F. asiaticum* was predominant, although its frequency was lower (85.6%). The fact that *F. asiaticum* dominated both populations indicates that it can circulate in the wheat-rice rotation system.

**Figure 2:**
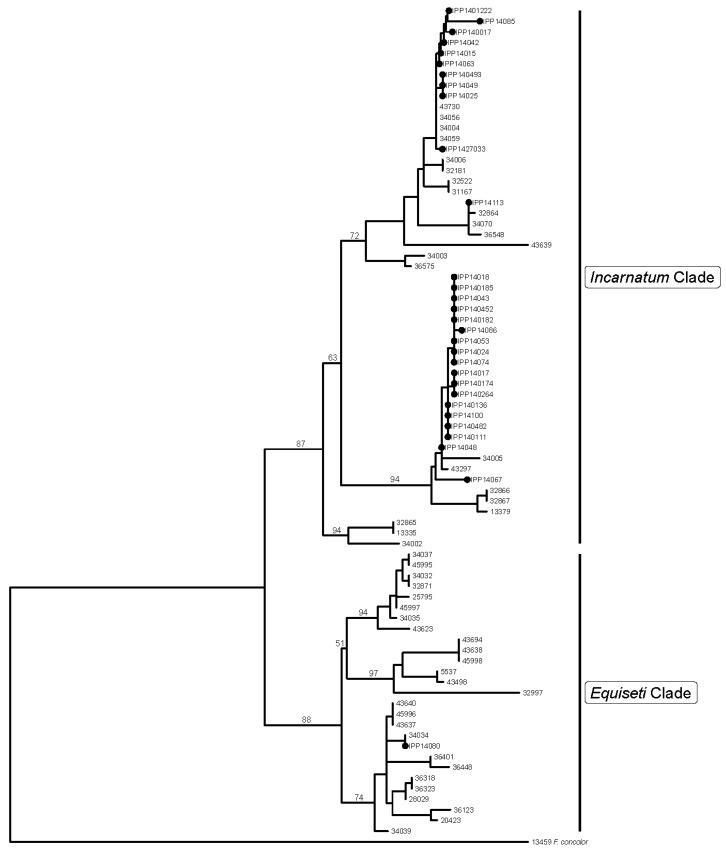
Maximum likelihood tree inferred from *TEF-1α* sequences by PhyML in Geneious 9.1. Strains labeled by a black dot were sequenced in this study, others are reference strains from O’Donnell et al., 2009. *F. concolor* (NRRL 13459) was set as an outgroup. Numbers above the branches are bootstrap values.

The majority of *F. graminearum* isolates 86.1% (55/67) were isolated from Sichuan Province; *F. graminearum* is rarely found in the provinces located in the middle and lower reaches of the Yangtze River. All nine *F. meridionale* strains collected originated from Sichuan Province and exhibited a significantly (Fisher’s Exact Test, *p* < 0.001) uneven distribution among rice and wheat, as only one isolate originated from wheat and eight from rice. Most non-FGSC species are at very low frequency except FIESC, which accounted for 50% of them (30/60). FIESC isolates were identified in all five provinces but were most commonly found on rice stubble (27/30). Phylogenetic analysis of FIESC showed that almost all isolates (29/30) belong to the *Incarnatum* clade with the exception of one isolate (IPP 14080) that belonged to the *Equiseti* clade (Figure 2).

### 2.2. Trichothecene Chemotype Identification

Trichothecene chemotypes of all 1186 FGSC isolates were identified by MLGT. The results are shown in Table 2. All *F. graminearum* and *F. meridionale* isolates are 15ADON and NIV producers, respectively. In the predominant species, *F. asiaticum*, all three chemotypes were found. Strains producing NIV (96.6%) dominated in Sichuan Province, especially on rice, where no other chemotypes were found. 3ADON is the main chemotype (80.5%) in the Hubei, Anhui, and Jiangsu Provinces, which are located in the middle and lower reaches of the Yangtze River. However, we found an uneven distribution of chemotypes in rice and wheat populations in these provinces. Significantly higher ratios (Fisher’s Exact Test, *p* < 0.001) of NIV producers were observed on rice stubble (20–35%) than on wheat kernels (7–10%) in these provinces (Figure 1B). In the nursery in Fujian Province, NIV was the main chemotype with the ratio of 64.0%. It is interesting that in Fujian Province up to 28.9% of *F. asiaticum* isolates were 15ADON producers, which was rare in other provinces we tested. 3ADON-producing *F. asiaticum* were only found in the wheat population with a low frequency (7.1%).

### 2.3. Pathogenicity Analyses

**Figure 3:**
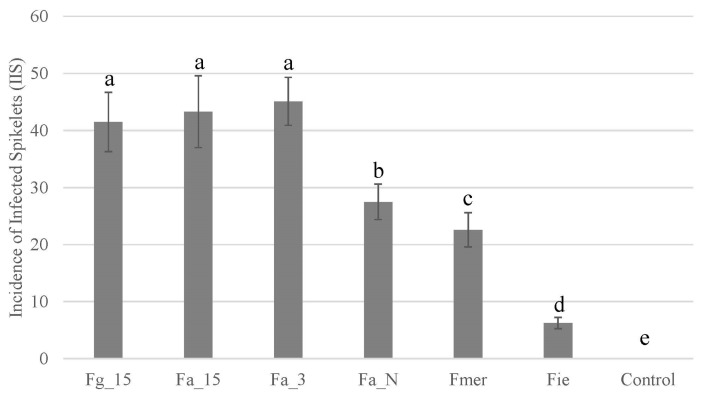
Pathogenicity of different *Fusarium* populations on wheat. Fg_15: 15ADON-type *F. graminearum*; Fa_15: 15ADON-type *F. asiaticum*; Fa_3: 3ADON-type *F. asiaticum*; Fa_N: NIV-type *F. asiaticum*; Fmer: *F. meridionale*; Fie: FIESC.

In order to understand how the hosts (wheat or rice) could influence their distribution of Fusarium species chemotype combinations, we selected 10 strains of each population including *F. graminearum*, *F. meridionale*, and FIESC as well as NIV, 3ADON, or 15ADON producers of *F. asiaticum* for pathogenicity analyses. Ten strains were selected randomly from each population. The three populations of *F. graminearum* (15ADON), *F. asiaticum* (3ADON), and *F. asiaticum* (15ADON) showed the highest incidence of infected spikelets (IIS), at 41.5 ± 5.2, 43.3 ± 6.3, and 45.1 ± 4.2, respectively. NIV-type *F. asiaticum* strains were clearly less aggressive (27.5 ± 3.1), but their pathogenicity was still significantly higher than that of *F. meridionale* isolates (22.6 ± 3.0), while FIESC strains showed the lowest IIS (6.3 ± 1.0), as most isolates in this population only caused symptoms in the inoculated spikelet which never extended to adjacent spikelets (Figure 3).

## 3. Discussion

FHB is one of the most important problems in wheat production worldwide. In the last decade, FHB outbreaks became much more frequent in China, where the epidemic area covered over 4 million ha in seven of 10 years. The popularization of straw-returning instead of burning of the preceding crops was suggested as the main reason, as it leads to increased numbers of pathogen propagules in the field. In this study, we surveyed the *Fusarium* composition on wheat kernels and on rice stubble in the same locations in five provinces of Southern China. The rice populations revealed higher species diversity than the wheat populations. *F. asiaticum* dominated both pathogenic and saprophytic populations, and there is no significant difference (*p* = 0.29, Fisher’s Exact Test) between the ratio of *F. asiaticum* in the two populations. This indicated that rice stubble may be selective for *F. asiaticum* rather than for other FHB species. It also confirmed that the perithecia on rice stubble are likely to act as the primary inoculum for FHB of wheat in Southern China. Del Ponte et al. (2015) compared the *Fusarium* composition on wheat heads and on corn residue in Brazil and observed a significant difference in two places; while *F. meridionale* dominated the saprophytic stage, *F. graminearum* was predominantly found in the pathogenic stage [14]. This may be due to the lower pathogenicity of *F. meridionale* on wheat—although they may show better fitness on corn residue (saprophytic stage), they failed in the competition with *F. graminearum* on wheat (pathogenic stage). In this study, we found similar pathogenicity levels in *F. asiaticum* with the 3ADON genotype and *F. graminearum* with the 15ADON genotype. We thus hypothesize that the preference of rice stubble in the saprophytic stage is crucial. Most *F. graminearum* strains (55/67) were isolated from Sichuan Province, which is consistent with our previous report [2]. In this region, although rice is prevalent, maize cultivation is also popular, which might explain the higher frequency of *F. graminearum* as *F. graminearum* is prevalent on maize.

In agreement with previous studies in China [2,21,22,23], we found *F. asiaticum* with the 3ADON genotype to dominate the Middle-Lower Yangtze River Plain, which is the area most frequently affected by FHB epidemics. However, NIV-producing *F. asiaticum* isolates were reported to be associated with almost all rice agroecosystems outside China. This difference may be caused by the selection on wheat. A strict wheat-rice rotation within one year is unique to this large area in China. Consequently, the *Fusarium* population is forced to go through two selection cycles, one on rice and one on wheat every year. In agreement with previous studies [2,24], we found that the pathogenicity of *F. asiaticum* with 3ADON and *F. graminearum* with 15ADON was significant higher than that of *F. asiaticum* with the NIV genotype on wheat (Figure 3). The better fitness of *F. asiaticum* on rice and DON producers on wheat leads to the prevalence of 3ADON-producing *F. asiaticum* in most wheat-rice rotation areas in Southern China. The observed chemotype composition supports this conclusion, as the ratio of 3ADON producers in wheat is significantly higher (*p* < 0.001, Fisher’s Exact Test) than that on rice in Hubei, Anhui, and Jiangsu, while in Sichuan and Fujian, 3ADON producers were only isolated on wheat but not on rice (Figure 1B, Table 2). In the absence of selection pressure by wheat, NIV-producing *F. asiaticum* dominates the population on rice seed in rice growing areas in South Korea [12] and Brazil [15]. Small populations of *F. asiaticum* with the NIV genotype were found on wheat that was grown near rice-growing areas in United States [13] and Brazil [14], and the inoculum was speculated to come from adjacent rice fields, possibly causing less frequent infections because of their lower virulence compared with DON-producing *F. graminearum*. In Eastern Uruguay, NIV-type *F. asiaticum* and 15ADON-type *F. graminearum* showed similar ratios (52% vs. 43%) in Cerro Largo, which is a new wheat production zone in traditionally rice growing areas. This can be explained by assuming that NIV-producing *F. asiaticum* isolates represent an old FHB population that existed before wheat was implemented, while the replacement by 15ADON-producing *F. graminearum* is still in progress in the new wheat production zone. Monitoring the species and chemotype shift of FGSC in this region could further validate our hypothesis and may be important for food safety because of the differences in toxicity of the trichothecenes.

Different from the provinces in the Middle-Lower Yangtze River Plain, the less virulent NIV population dominated both wheat and rice populations in Sichuan Province, which is traditionally a wheat-rice rotation region. In Sichuan Province isolates with the 3ADON chemotype were only isolated on wheat and at low frequency (9.5%). This may be due to the decrease of the wheat production in Sichuan. The mountain region in this province restricts the use of machines and, along with the increase of the labor costs, more and more farmers have abandoned wheat cultivation. Consequently, the selection pressure for more virulent species on wheat also decreased. In addition, the mountains and the small holder farming in this region also limits the spread of successful populations on wheat. In Japan, where wheat cultivation is rare and rice production occurs across the entire country, 70% of the *F. asiaticum* isolates on wheat are NIV-type, much higher than 3ADON producers (29%) [6]. Also, in this case the lack of selection on wheat could be responsible for the prevalence of the *F. asiaticum* NIV producers. In Fujian, the situation is distinct from other provinces in this study because farmers abandoned wheat cultivation due to severe FHB epidemics more than 10 years ago. In the present study, we could only collect samples in a small nursery dedicated to evaluating FHB resistance of wheat varieties from other provinces. NIV-producing *F. asiaticum* dominated both wheat and rice populations and only a few 3ADON producers were found on wheat, which may be introduced by wheat seeds from the Middle-Lower Yangtze River Plain. The presence of abundant NIV-type *F. asiaticum* in non- (Fujian) and less-wheat (Sichuan) provinces revealed that not only wheat selects 3ADON producers, but also that rice agroecosystems favor NIV producers. This strong association between NIV producers and rice is in agreement with previous reports from Korea [12], Japan [6], the United States [13], Uruguay [11], and Brazil [15], further underpinning this conclusion. Additional evidence was found in the Middle-Lower Yangtze River Plain, where in all three provinces significantly higher ratios of NIV producers were observed on rice stubble (20–35%) than on wheat kernels (7–10%), despite the fact that 3ADON is the main chemotype in this region (Figure 1B, Table 2). In agreement with previous reports [23,24], we also found a high ratio of 15ADON-producing *F. asiaticum* on both rice and on wheat in Fujian, which is rare in wheat growing areas. This population showed a similar level of pathogenicity to 3ADON-producing *F. asiaticum* and 15ADON-producing *F. graminearum* on wheat, but higher aggressiveness than NIV producers of *F. asiaticum* and *F. meridionale* (Figure 3).

All nine *F. meridionale* isolates collected originated from Sichuan. Previous studies reported the presence of *F. meridionale* in Sichuan and Yunnan on wheat [2] and Sichuan and southern Gansu on maize [19] in China. Similar to our study, it was typically found in low frequencies. Based on the reports so far, it seems that this species is limited to southwest China. *F. meridionale* has been reported as the main species on maize in Nepal and in Northern Argentina [25,26]. Small populations of *F. meridionale* were also found in Korea [7] and in South Africa [18]. Recently, a large *F. meridionale* population was identified on wheat in Brazil, which was thought to be related to the two harvests of maize in one year [14]. *F. meridionale* was also shown to be less competitive on wheat than *F. graminearum* isolates [14]. Similar results were obtained in this study, as we found *F. meridionale* to be less virulent than both *F. graminearum* and *F. asiaticum* on wheat. This may be the main reason for the uneven distribution of *F. meridionale* on wheat (1/9) vs. rice stubble (8/9).

In addition to FGSC, 60 non-FGSC *Fusarium* isolates where obtained and half of these strains were identified as FIESC. Among them, three isolates were found on wheat while the other 27 were isolated from rice stubble. In the pathogenicity assays, most FIESC isolates caused only symptoms on the inoculated spikelets but no spreading was observed, which may explain the absence of this species in the main wheat producing regions. Phylogenetic analysis showed that 29 strains were clustered in the *Incarnatum* clade and the remaining one in the *Equiseti* clade. Lineages in the *Incarnatum* clade are not known to have a sexual stage [27]. However, the rice stubble population in our study was obtained as single spores isolated from ascospores (29 strains from five provinces). Therefore, we hypothesize that a natural sexual cycle occurs on rice and it may be important to study this sexual cycle and its consequences for population diversity and epidemiology in more detail.

Many previous studies on the occurrence and prevalence of FGSC exist and the composition of populations is reported to change over time [28]. However, the drivers for this phenomenon are still largely unknown. Recently, based on available data from the literature, we concluded that a cropping system with wheat/maize rotation selects for *F. graminearum*, while a wheat/rice rotation selects for *F. asiaticum*. *F. boothii* is selected when maize is cultivated without rotation [19]. In this study, we further investigated the *Fusarium* population on rice stubble on a large scale and made comparisons with populations from wheat kernels. We confirmed that perithecia on rice stubble were the primary inoculum for FHB on wheat. Based on our findings and previous reports across China, we conclude that wheat is selective for DON-producing *F. graminearum* and *F. asiaticum* due to higher pathogenic fitness. In contrast, rice favors *F. asiaticum* with the NIV chemotype, possibly due to increased saprophytic fitness. Depending on the cropping system, this divergent selection can shape *Fusarium* populations and this can help us to predict possible prevalent species and subsequently predict which mycotoxin contamination can be expected.

## 4. Materials and Methods

### 4.1. Fungal Isolates

Rice stubble with black perithecia was collected from nine sampling sites in five provinces (Figure 1A) in Southern China from 30 March to 7 April 2014. Sichuan, Hubei, Anhui, and Jiangsu Provinces are common wheat producing areas and wheat-rice rotation is the most prevalent cropping system. However, there is no wheat grown in Fujian Province, and the sampling site Nanping is just a small nursery where wheat is rotated with rice. Sichuan Province is a mountainous area, located in the upper valley of the Yangtze River. Hubei, Anhui, and Jiangsu Provinces are located in the Middle-Lower Yangtze River Plain. Diseased wheat spikes with mature kernels were sampled in the same place from 27 April to 10 May 2014. For wheat samples, diseased kernels were surface-sterilized in 70% ethanol for 30 s, and immediately immersed in 2% sodium hypochlorite for 90 s, after which the kernels were extensively rinsed with sterile distilled water and placed on potato dextrose agar (PDA) plates. After 3 days of incubation at 26 °C, newly grown-out mycelium was transferred into flasks containing 20 mL of mung bean broth cultures (3% mung bean extract). Flasks were shaken at 180 rpm for 2 days at 26 °C. Spore suspensions were diluted to 100-fold and plated on 1% water agar. The plates were incubated for 12 to 24 h and single spores were identified and transferred to PDA plates. Single spore cultures were stored in 15% dimethyl sulfoxide (DMSO) at −80 °C. For rice samples, the rice stubble with perithecia were excised and surface-sterilized as described above. Small pieces of perithecia were picked up and soaked in 100 μL sterile distilled water in PCR tubes. After 6–8 h incubation at room temperature, ascospores were allowed to be released in the water. Subsequently, single spore isolations were performed as described above. Strain information is provided in Table S1.

### 4.2. Genomic DNA Extraction

A small mycelial plug was transferred to 6-cm Petri plates containing potato dextrose agar (PDA) and incubated at 26 °C for 5 days. Mycelium was harvested with scalpel blades and vacuum freeze-dried at −20 °C overnight. The mycelium was lyophilized using liquid nitrogen and grinding by vigorous shaking in a MiniBeadbeater-96 (Biospec, Bartlesville, OK, USA). Total genomic DNA was extracted using the SP Fungal DNA Kit (Omega Biotek, Norcross, GA, USA) according to the manufacturer’s instructions. DNA concentration was determined by NanoVue Plus (GE, Pittsburgh, PA, USA). Finally, DNA samples were diluted to 5 ng/mL for each sample in 96-well microtiter plates, which were stored at −20 °C.

### 4.3. Species and Trichothecene Chemotype Determination

Multilocus genotyping (MLGT) assay [10] was used to determine the species and trichothecene chemotype of all strains. For the isolates that could not be identified by MLGT, partial translation elongation factor (*TEF-1α*, 700 bp) gene sequences were generated with the previously reported primers [29]. These strains were identified by sequence comparison in the Fusarium MLST database [30] and/or in GenBank. Based on the *TEF-1α* sequence, a maximum likelihood tree of FIESC isolates together with several reference strains (from O’Donnell et al., 2009) was constructed in Geneious 9.1 using PhyML, and *F. concolor* (NRRL 13459) was set as an outgroup.

### 4.4. Pathogenicity Analyses

A medium resistant winter wheat cultivar, Yangmai 158, was used for the pathogenicity tests. Wheat was planted in three different blocks (2 m × 4 m) according to normal agronomic practices at the Langfang Farm (Experiment Farm of the Institute of Plant Protection) in 2015. We selected 10 strains from each population randomly (nine isolates from *F. meridionale* population) for pathogenicity analyses. Conidia were prepared by mung bean broth cultures as mentioned above. At anthesis, 10 heads in each block were inoculated by injecting 20 mL conidial suspension (10^6^ conidia/mL) of one isolate into a central floret of a spike (injection inoculation). A total of 30 heads was infected by a single strain. Ten heads in each block were also selected to be inoculated with 20 μL sterilized water as a control group. Pathogenicity was assessed as the incidence of infected spikelets, which was determined visually by counting the number of infected spikelets per head at 14 days after inoculation and expressed as a percentage of the total number of spikelets.

## Figures and Tables

**Table 1:** Distribution of *Fusarium* isolates from wheat and rice in Southern China.

Species Complex *	Provinces	Sichuan						Hubei		Anhui				Jiangsu				Fujian		Total
	Sampling Sites	Jianyang		Guanghan		Mianyang		Xiaogan		Fengtai		Xuanzhou		Nanjing		Yizheng		Nanping		
	Hosts **	W	R	W	R	W	R	W	R	W	R	W	R	W	R	W	R	W	R	
FGSC	*F. asiaticum*	17	56	38	49	28	74	57	60	42	28	50	81	28	75	37	65	211	114	1110
	*F. graminearum*	4	21	1	1	14	14	0	0	1	1	0	1	0	1	2	0	6	0	67
	*F. meridionale*	1	5	0	1	0	2	0	0	0	0	0	0	0	0	0	0	0	0	9
FIESC		0	0	0	1	0	0	0	21	0	0	0	2	0	0	0	1	3	2	30
FTSC	*F. acuminatum*	0	0	0	0	0	0	0	1	0	0	0	0	0	0	0	0	0	0	1
	*F. tricinctum*	0	0	0	0	0	0	0	1	0	0	0	0	0	0	0	0	0	0	1
FSPSC	*F. armeniacum*	0	0	0	0	0	0	0	0	0	0	0	6	0	0	0	0	0	0	6
FSAMSC	*F. kyushuense*	0	0	0	0	0	0	0	1	0	0	0	0	0	0	0	0	1	0	2
FFSC	*F. fujikuroi*	0	0	0	0	0	0	0	0	0	0	0	0	1	0	0	6	0	0	7
	*F. proliferatum*	0	2	0	0	0	0	0	0	0	0	0	0	0	0	0	4	0	0	6
	*F. concentricum*	0	3	0	0	0	0	0	0	0	0	0	0	0	0	0	0	0	0	3
	*F. nygamai*	0	0	0	0	1	0	0	0	0	0	0	0	0	2	0	0	0	0	3
	*F. verticillioides*	0	0	0	0	0	0	0	0	0	0	1	0	0	0	0	0	0	0	1
	Total	22	87	39	52	43	90	57	84	43	29	51	90	29	78	39	76	221	116	1246

* Species complexes: FGSC, *F. graminearum* species complex; FIESC, *F. incarnatum-equiseti* species complex; FTSC, *F. tricinctum* species complex; FSPSC, *F. sporotrichioides* species complex; FSAMSC, *F. sambucinum* species complex; and FFSC, *F. fujikuroi* species complex. ** W represents wheat and R represents rice.

**Table 2:** Trichothecene type compositions of FGSC isolates.

Hosts	Provinces	*F. asiaticum*			*F. graminearum*	*F. meridionale*	Total
		NIV	3ADON	15ADON	15ADON	NIV	
Wheat	Sichuan	74	8	1	19	1	103
	Hubei	4	50	3	0	0	57
	Anhui	10	81	1	1	0	93
	Jiangsu	5	60	0	2	0	67
	Fujian	147	23	41	6	0	217
Rice	Sichuan	179	0	0	36	8	223
	Hubei	21	36	3	0	0	60
	Anhui	31	78	0	2	0	111
	Jiangsu	28	108	4	1	0	141
	Fujian	61	0	53	0	0	114
	Total	560	444	106	67	9	1186

## Supplementary Materials

The following are available online at http://www.mdpi.com/2072-6651/10/3/115/s1, Table S1: Strains information in this study.
